# Estimating Free-Energy Surfaces and Their Convergence from Multiple, Independent Static and History-Dependent Biased Molecular-Dynamics Simulations with Mean Force Integration

**DOI:** 10.1021/acs.jctc.4c00091

**Published:** 2024-06-24

**Authors:** Antoniu Bjola, Matteo Salvalaglio

**Affiliations:** Thomas Young Centre and Department of Chemical Engineering, University College London, London WC1E 7JE, U.K.

## Abstract

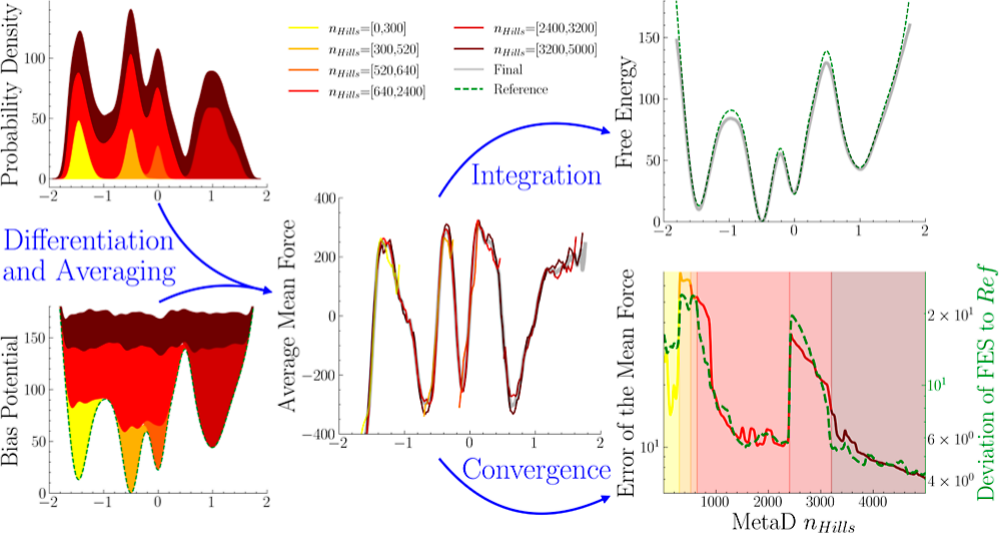

Addressing the *sampling problem* is central to obtaining quantitative insight from molecular dynamics simulations. Adaptive biased sampling methods, such as metadynamics, tackle this issue by perturbing the Hamiltonian of a system with a history-dependent bias potential, enhancing the exploration of the ensemble of configurations and estimating the corresponding free energy surface (FES). Nevertheless, efficiently assessing and systematically improving their convergence remains an open problem. Here, building on mean force integration (MFI), we develop and test a metric for estimating the convergence of FESs obtained by combining asynchronous, independent simulations subject to diverse biasing protocols, including static biases, different variants of metadynamics, and various combinations of static and history-dependent biases. The developed metric and the ability to combine independent simulations granted by MFI enable us to devise strategies to systematically improve the quality of FES estimates. We demonstrate our approach by computing FES and assessing the convergence of a range of systems of increasing complexity, including one- and two-dimensional analytical FESs, alanine dipeptide, a Lennard–Jones supersaturated vapor undergoing liquid droplet nucleation, and the model of a colloidal system crystallizing via a two-step mechanism. The methods presented here can be generally applied to biased simulations and are implemented in *pyMFI*, a publicly accessible, open-source Python library.

## 1 Introduction

Molecular dynamics (MD) simulations have become a powerful tool for studying and predicting the dynamics and thermodynamics of molecular systems. They allow scientists to develop insight into the collective behavior of complex systems at the atomistic scale. The quantitative assessment of equilibrium properties in molecular systems and their interpretation is associated with estimating free-energy surfaces (FES) as a function of a low-dimensional set of collective variables (CVs), **s**. An FES provides information on a molecular system of interest by quantifying the equilibrium probability of ensembles of configurations corresponding to relevant states and providing information on low-energy transition pathways. However, molecular systems are often characterized by multiple metastable states, separated by high free-energy barriers. This renders transitions between states rare and the sampling necessary to converge thermodynamic properties computationally inaccessible. Numerous methods have been proposed to overcome the sampling problem and enhance the exploration of configuration spaces despite the presence of high-energy barriers.^1−5^

A subclass of these methods is based on perturbing the system Hamiltonian via an opportunely defined bias potential, which allows for efficient exploration of the relevant configuration space. Multiple approaches belong to this class.^3,4,6−9^ Among these, two widely used methods are Umbrella sampling (US)^4,10^ and metadynamics (MetaD).^3,7^ These two methods exemplify two opposite and complementary ways of defining a potential to enhance the sampling of rare transitions. US introduces multiple, independent replicas—often referred to as *windows*—in which a time-independent, harmonic bias potential defined in CV space is introduced to localize the sampling on a specific set of configurations. On the other hand, MetaD, as well as other adaptive sampling methods,^11,12^ introduces a history-dependent bias potential that evolves dynamically with the system and, in the long-time limit, provides an estimate of the free energy in the CV space explored. While in US a global FES is obtained by merging the sampling obtained in each window with algorithms such as the weighted histogram analysis method^6,13^ or Umbrella integration (UI),^14−16^ MetaD provides a global FES directly as a function of the cumulative bias potential,^3,7,8^ or via reweighting.^9,17−20^ However, since static bias approaches such as US are based on independent windows, they are trivially parallel and enable a systematic sampling augmentation to reduce the uncertainty of the reconstructed FES. On the other hand, MetaD offers an autonomous exploration of the configuration space, essential when pathways connecting metastable states in CV space are unknown a priori.

Recently, we demonstrated that a single global FES can be obtained from multiple independent asynchronous MetaD replicas by mean force integration (MFI).^21^ Here, we build on this result to develop a systematic approach to combine the information obtained from sampling phase space with multiple independent MD simulations performed under the effect of various biases, both static and history-dependent. For instance, using MFI to reconstruct an FES from independent US and MetaD simulations opens up the possibility of systematically improving the uncertainty of FESs by sampling poorly converged regions. In addition, based on the MFI formalism, we develop a systematic estimation of local and global convergence of the target FES that can be computed on-the-fly and applied to multiple asynchronous replicas subject to different biasing methods. Such measures can be used to inform and systematically improve free-energy estimates by concentrating the sampling in poorly converged regions of CV space.

## 2 Theoretical Background: MFI

The FES *F*(**s**), as a function of a low-dimensional set of CVs **s**, in the presence of a bias potential defined in CV space *V*(**s**), can be expressed as^6,14,21^
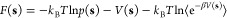
1where *k*_B_ is the Boltzmann constant, *T* is the temperature, and *p*(**s**) is the biased probability density sampled under the effect of the bias potential *V*(**s**). The term  is the reversible work associated with the introduction of the bias potential *V*(**s**) in the unperturbed ensemble.

Inspired by UI,^1,14−16^ MFI relies on the calculation of the mean thermodynamic force in **s**, −∇_**s**_*F*(**s**). The advantage of computing the gradient of the free energy in **s**, instead of directly estimating *F*(**s**), lies in the fact that the former does not require the estimate of the term . In fact, such a term is independent of **s** and represents a constant offset of the free energy in eq 1. As a consequence, the mean force −∇_**s**_*F*(**s**) can be estimated from simulations performed under the effect of different bias potentials without evaluating alignment constants between samples obtained under the effect different biases. We note that several techniques have been developed to obtain such constants for simulations evolving under the effect of static^13,22^ and MetaD^9,17−20^ biases.

By focusing on the derivative of the free energy, MFI^21^ is similar in spirit to force-based methods for the calculation of probability densities from atomistic data,^23,24^ which have been shown to converge to a smoother estimate of the probability density and have been recently used in conjunction with MetaD to accelerate the convergence of FES estimates from ab initio MetaD calculations.^25^

Moreover, as shown by Awasthi et al.,^26^ combining US and WTmetaD, that is, static and history-dependent biases, can be beneficial in several use cases. MFI provides an alternative, flexible framework for combining multiple independent simulations carried out asynchronously with different biasing approaches, thus enabling the systematic refining of FES estimates by mixing data obtained with different sampling protocols.

### 2.1 MetaD with MFI

In this section, we summarize the main features of MFI applied to obtain an FES from MetaD in a monodimensional CV space *s* to provide a basis for introducing additional bias potentials and mean force convergence estimators. For history-dependent biasing methods such as MetaD, the simulation time is divided into time windows of constant bias denoted by subscript *i*. Without loss of generality, the mean force of time-window *i*, , in a monodimensional CV space *s*, can be written as^10,14,21^
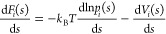
2where *p*_*i*_(*s*) is the bias probability density, sampled during a time-window *i* under the effect of a constant bias potential *V*_*i*_(*s*). In ref (21), it has been shown that this expression holds in the case of a MetaD bias potential updated in *discrete* time-steps.

The average mean force obtained after an arbitrary number of *N* time-windows can be obtained as a weighted average of , with weights proportional to *p*_*i*_(*s*)
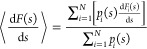
3Both eq 3 and the first term in eq 2 require an estimate of the bias probability density, which is constructed from configurations sampled during some time-window *i*. The time-window *i* starts at time *t*_*i*_, when the system is first sampled under the effect of a new (updated) bias potential *V*_*i*_(*s*) and ends with the last sample under the effect of that bias at time *t*_*i*_ + τ, where τ is the time between two consecutive updates of the bias potential. Thus, the bias probability density of time-window *i* is estimated as a sum of Gaussian kernels
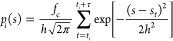
4where *h* is the bandwidth of the Gaussian kernel and *f*_c_ is the height, which corresponds to the sampling rate of configurations. The term *f*_c_/*h* serves as a scaling factor that facilitates the combination of bias probability densities that employ different *h* or *f*_c_. This choice leads to the following expression of the first term in eq 2
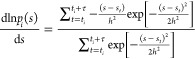
5

The second term in eq 2 is straightforward. It represents the contribution to the mean force associated with the bias potential *V*_*i*_(*s*) and is computed as the derivative of the MetaD potential accumulated up to time-window *i* as a sum of Gaussian kernels centered in *s*_*i*_, with height *w*_*i*_, and width σ_M,*i*_
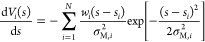
6

By combining eqs 2–6, the unbiased average mean force for *N* consecutive time-windows is estimated in a closed analytical form.^21^ The resulting unbiased average mean force  is then integrated numerically to obtain the FES *F*(*s*). In analogy with UI, obtaining *F*(*s*) by integrating the mean force, while general, is practically limited by the dimensionality of the CV space used to define the bias potential, and therefore, as UI, MFI is practically applicable for CV spaces with dimensionality ≤3. Moreover, integration can introduce numerical errors in the calculation of *F*(*s*). As discussed in detail by Kästner in ref (16) for UI, such errors are significantly smaller than inherent sampling errors. This is empirically observed in all the analytical potentials studied in this work, where the MFI estimate of *F*(*s*) achieves vanishingly small deviations from the analytical free energies. Finally, integration accuracy and efficiency depend on the algorithm adopted and on the density of the chosen integration grid. In ref (21), we show convergence for increasingly dense grids using finite-differences integration methods. Here, we obtain analogous results by adopting either finite-differences^21^ or a fast Fourier transform integration.^16^

### 2.2 Combining Independent Simulations

As mentioned above, the strength of MFI is the possibility of combining the sampling obtained from *independent* and *asynchronous* simulations. This can be done by appropriately extending the weighted average given by eq 3 to include the mean forces of multiple independent simulations. The resulting equation combines the average mean forces ⟨d*F*(*s*)/d*s*⟩_*j*_ of simulations *j*, with a weight corresponding to the biased probability density of that simulation . The combined average mean force over *M* independently biased trajectories can thus be determined using a weighted average analogous to eq 3, as
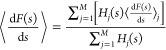
7

Combining independent simulations in this fashion opens the door to asynchronous MetaD simulation campaigns that may optimize the use of computational resources but also allow for local refinement of the sampling of configurations.

In the following, we discuss how, within the framework of MFI, one can estimate the convergence of average mean force and the FES to identify regions of configuration space that require additional sampling. Later, we lay out methods to combine static and history-dependent bias potentials to systematically improve the sampling as well as parallel and serial simulation structures. We do so by demonstrating these features for Langevin models in mono- and bidimensional CV spaces, for the *ever-present* alanine dipeptide and for a more challenging collective process of liquid droplet nucleation from a supersaturated argon vapor and for a colloidal crystal nucleating from solution. The methods and analyses reported here are implemented in *pyMFI*, a publicly accessible Python library available at https://github.com/mme-ucl/pyMFI. Example scripts using *pyMFI* are reported in the Supporting Information.

## 3 Convergence

With bias-enhanced sampling simulations, practitioners typically pursue two concurrent objectives. The first is the exploration of relevant configurations that are rarely sampled during standard MD simulations. The second is the estimate of the equilibrium probability of such configurations. History-dependent biased sampling methods combine these two objectives by evolving under the effect of a bias potential that encourages the autonomous exploration of configuration space and that enables the estimate of the equilibrium probability of appropriately defined sets of configuration. Therefore, a useful convergence metric for biased sampling simulations should acknowledge these two complementary aspects by accounting for the uncertainty in the FES calculation and for the extent of the sampling. Typically, the convergence of the FES is estimated using block averaging techniques,^27,28^ and error propagation.^29^ This approach is carried out *a posteriori*, often requiring the assumption of a time-independent bias, even though time-dependent reweighting techniques can also be used.^7,18−20^

### 3.1 On-the-Fly Assessment of Biased Sampling Convergence

With MFI, the time-independent average mean force ⟨d*F*(*s*)/d*s*⟩ is estimated as the running weighted average with eq 3. This implies that the uncertainty of the average can be estimated by computing the weighted variance^30,31^ of the mean forces of each time window, with weights proportional to the biased probability density *p*_*i*_(*s*) sampled in the respective time window.^10,14^ Employing the notation introduced so far, the variance of the average mean force can be expressed as
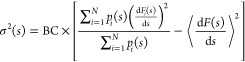
8where σ(*s*) is the weighted standard deviation, and BC represents the Bessel correction for the variance of the weighted mean, defined as
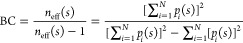
9where  is the effective sample size. The standard error of the weighted mean is expressed as
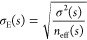
10

All the terms appearing in eqs 8–10 can thus be computed based on the history of the simulation up to time-window *i*. As such, the local estimate of the variance obtained from eqs 8–10 provides an *on-the-fly*, *local* measure of convergence of the mean thermodynamic force. By averaging σ_E_(*s*) value over the *sampled* CV space, we can obtain a global convergence estimator of the mean force: .

Furthermore, we note that  can only be evaluated in *explored* regions of *s* since in unexplored regions, the force is not determined. To develop a convergence metric incorporating both the uncertainty in the FES calculation and the extent of the configurational volume explored, we divide σ_E_ by *v*, a measure of the volume of CV space explored by the simulation. In this work, we express *v* as a nondimensional quantity by dividing the volume of the sampled configuration in CV Space by the total volume of the domain in CV space considered to be relevant for the problem studied. The former is evaluated as the volume of the CV domain where the biased histogram of configurations *H*_*j*_(*s*) is larger than a lower-bound arbitrary threshold.

**Figure 1:**
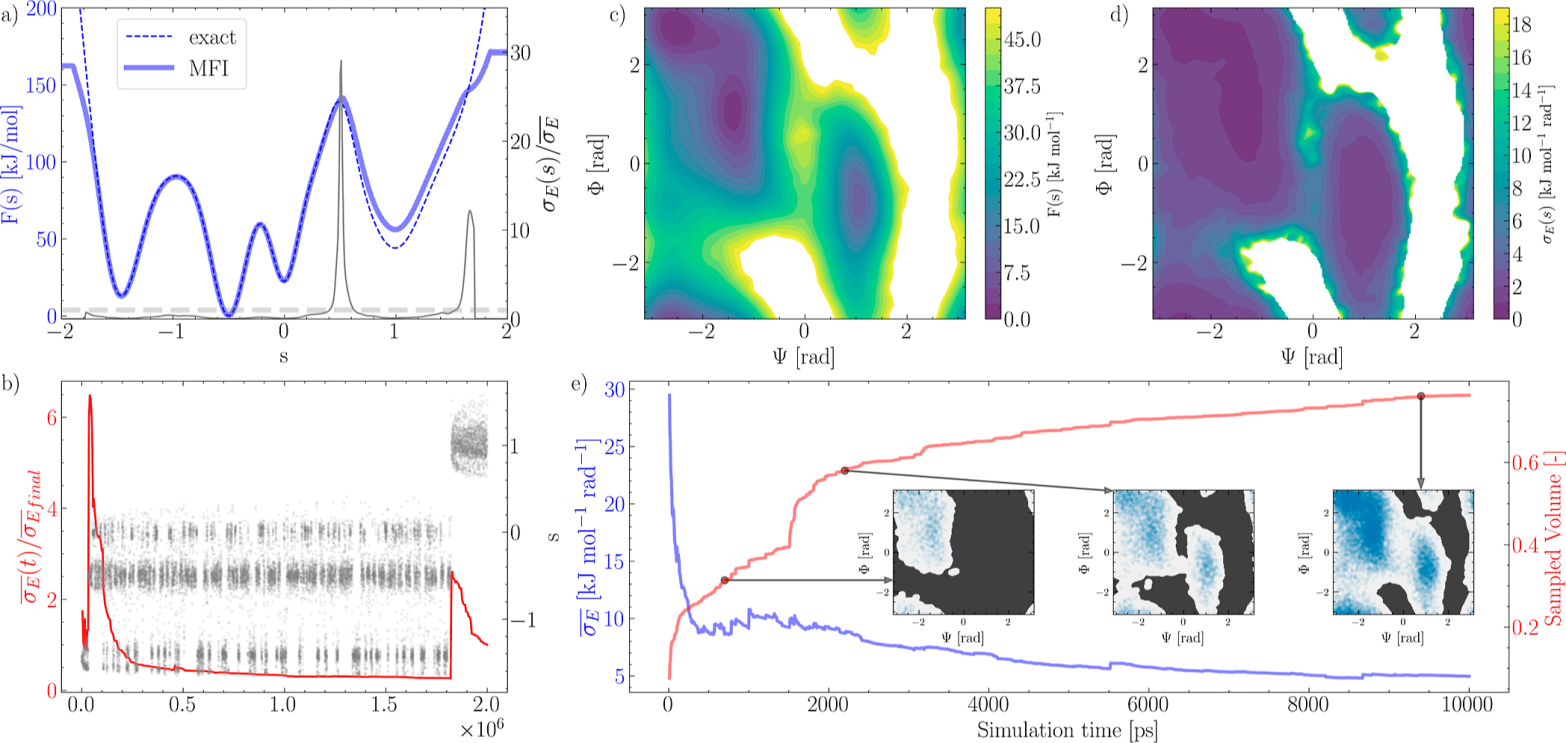
(a) Comparison between the MFI estimate of a monodimensional multiwell FES obtained postprocessing a single MetaD simulation (solid blue line) with the exact FES (dashed blue line), and the normalized standard error of the mean force,  (solid gray line), normalized by its average value over the sampled CV space,  (dashed gray line). (b) CV space exploration (gray symbols) and normalized average variance of the mean force (solid red line). (c) Alanine dipeptide FES, function of the Φ and Ψ dihedral angles. (d) Standard error of the mean force mapped in the Φ and Ψ torsional angles. (e) Average standard error of the mean force (blue) and ratio of configurational space explored (red) as a function of the simulation time.

The different components of the convergence estimator  are illustrated in Figure 1: panels a and c report the FES computed by integrating the average mean thermodynamic force computed with eq 3 for a monodimensional Langevin dynamics model evolving on an analytical potential defined as
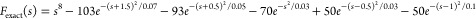
and for Alanine Dipeptide, a typical example of a two-dimensional FES.

In Figure 1a (in gray, right axis) and Figure 1d, we report for these two systems σ_E_(*s*), the standard error of the weighted mean mapped onto the CV space *s*. In Figure 1a, σ_E_(*s*) is normalized by its average value estimated over the entire *sampled* CV space, .

In Figure 1b, it can be seen that the dynamic evolution of  captures the overall convergence of the sampling in *s*. In particular, it can be seen that  relaxes rapidly, with the system reversibly sampling numerous transitions between the multiple local basins characterizing the FES for *s* < 0.5. Such relaxation is representative of the increasing confidence in the estimate of the local mean force. When the system *discovers* a new metastable state, for *s* > 0.5 at ≈1.7 × 10^6^ steps, the mean force in the newly discovered metastable state is characterized by a lower level of confidence, thus resulting in a sudden increase in .

A similar trend can be seen in Figure 1e, displaying the dynamic evolution of  of alanine dipeptide (blue, left axis) together with the total CV space sampled (red, right axis). At the start of the simulation, the low-energy region of the left basin is being sampled, causing an immediate decrease in the mean force error. As the height of the MetaD potential increases, higher energy regions are discovered, and  stops decreasing.

**Figure 2:**
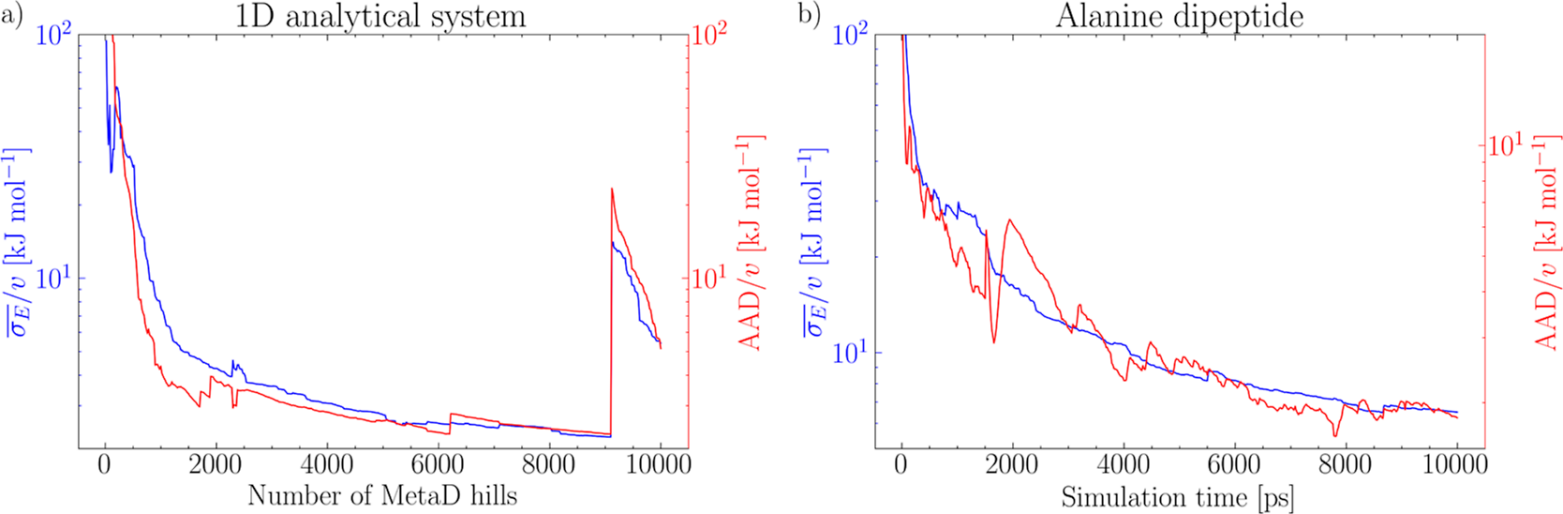
Comparing the dynamic evolution of the average error of the mean force normalized by the sampled volume (*left y*-axis) and the AAD normalized by the sampled volume (*right y*-axis). The former can be computed on-the-fly based on the history of the biased simulation without knowledge of an external reference FES. The latter can only be estimated only for toy examples with a known exact FES. (a) Multiwell one-dimensional FES (see Figure 1a). (b) Alanine dipeptide (see Figure 1c).

It should be noted that σ_*E*_(*s*), and  enable to assess the convergent behavior of the simulations but provide only a qualitative measure of the real error in the mean force due to correlations in the mean force that might be present at small sample sizes. Notably, however, the normalized convergence estimator  correlates strongly with the normalized average absolute deviation (AAD/*v*) of the FES from an independent reference, as shown in Figure 2a for the monodimensional FES and for alanine dipeptide in Figure 2b. This is an important observation as, while the AAD is arguably an objective and accurate measure of convergence, it cannot be computed for any realistic application, while  can be computed on-the-fly and enables a systematic assessment of FES convergence.

### 3.2 Convergence for Sets of Independent Biased Simulations

The measure of convergence discussed in the previous section can be naturally extended to cases where the FES is computed from multiple independent simulations by computing the variance of the time-averaged mean force over *M* independent simulations (see eq 7) as
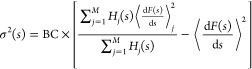
11where the Bessel correction now takes into account the total weight of each simulation via *n*_eff_(*s*) defined as
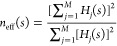
12

For such cases, the mean forces obtained from individual simulations are not correlated and can be used to estimate the FES error with a bootstrapping analysis.^28^ This is demonstrated in the first example of Section 5.

## 4 Combining Multiple Biases and Independent Simulations

The ability to estimate the local convergence of the mean force in CV space *on-the-fly* combined with the possibility of locally refining the sampling in specific regions of phase space by merging the information obtained from independent simulations through eq 7 suggests that to efficiently achieve local refinement of free energy estimates one may aim to apply different biasing strategies at different stages of exploration/convergence. In particular, to locally enhance the accuracy of FES estimates, it might be useful to combine static and time-dependent biases. By introducing *n*_B_ bias potentials that simultaneously act on a system, the mean force of a generic time-window *i* becomes
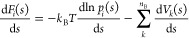
13where the term  can include any differentiable static bias. Typical choices that enable to focus the sampling in specific regions of the CV space of interest include harmonic potentials upper and lower walls, but static biases are not restricted to these cases.

To demonstrate the feasibility of combining different biases and using an on-the-fly convergence metric to monitor the behavior of the calculations, we investigate a two-dimensional double-well analytical potential often used in the enhanced sampling literature.^12,32^ The exact free energy for such model is defined as *F*_exact_(*s*_1_, *s*_2_) = 1.35*s*_1_^4^ + 1.90*s*_1_^3^*s*_2_+3.93*s*_1_^2^*s*_2_^2^ – 6.44*s*_1_^2^ – 1.90*s*_1_*s*_2_^3^ + 5.59*s*_1_*s*_2_ + 1.33*s*_1_ + 1.35*s*_2_^4^ – 5.56*s*_2_^2^ + 0.90*s*_2_ + 18.59.

### 4.1 Combining Multiple Short MetaD Simulations

As discussed in the introductory section, an advantage of MFI is the ability to construct an FES from multiple asynchronous MetaD simulations. This can be exploited by running simulations in a trivially parallel manner and increasing the sampling for configurations that, while having been visited by previous trajectories, are far from convergence. This provides additional flexibility for running MetaD simulations and allows for a more efficient use of computational resources.

To present this feature, we compare a long MetaD simulation with 20 short simulations totaling the same number of steps. The short MetaD simulations are performed under a set of parameters identical to the long simulation (see the Supporting Information for details) and are initialized in a random configuration. We note that, for atomistic examples, random reinitialization would only be possible within the set of configurations that have already been explored by existing trajectories. This is demonstrated and discussed in the Results section for the condensation of a LJ vapor, where many short simulations are initialized from the two known metastable states, that is, liquid droplet in vapor and supersaturated vapor, and in ref (33), where an analogous approach is used to study the free-energy landscape associated with reactive events.

**Figure 3:**
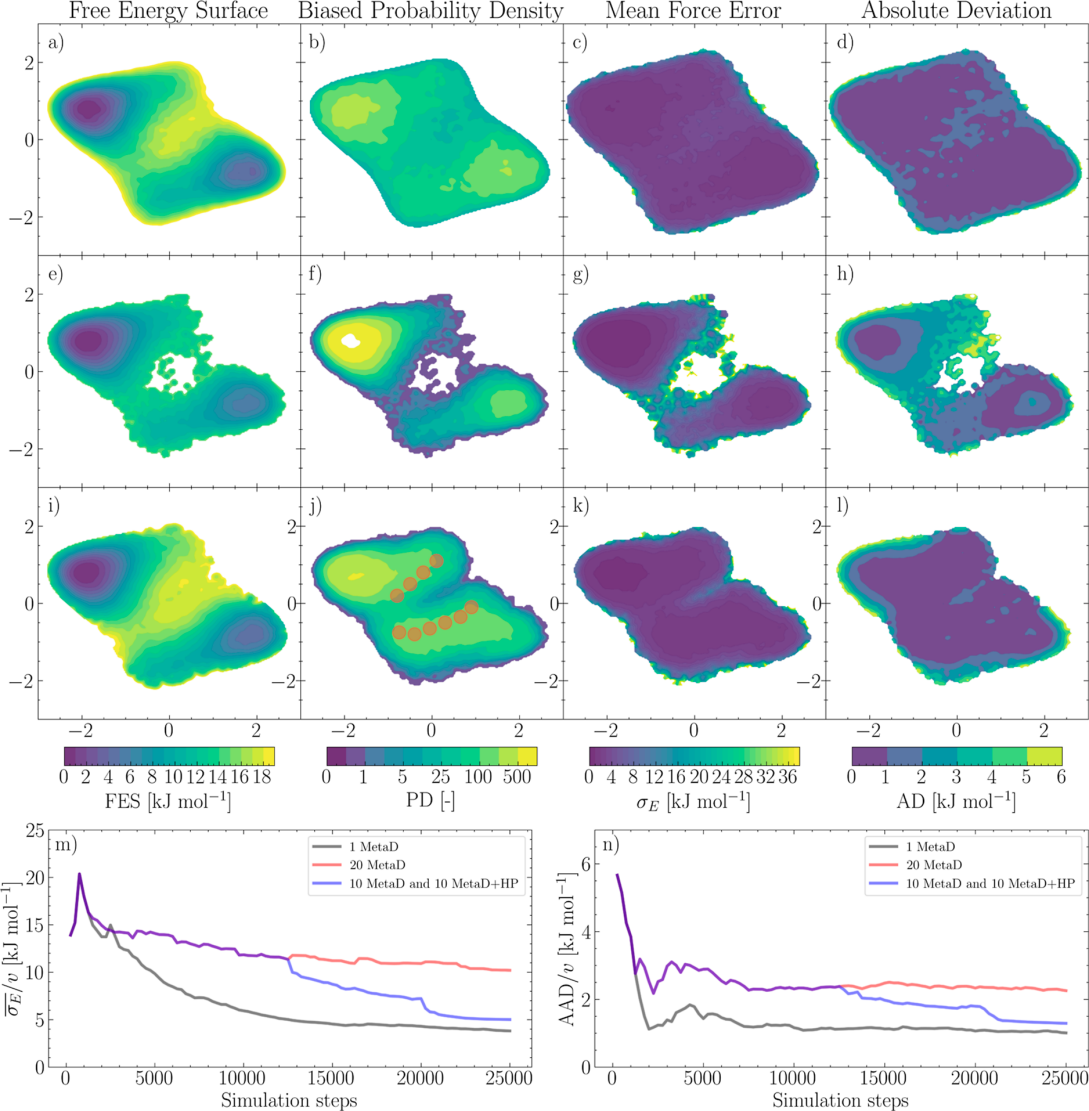
FESs from independent, biased simulations. FES, total biased probability density , CV map of the mean force error, and absolute deviation from the analytical FES for a two-dimensional double-well model potential. (a–d) Single, long WTmetaD simulation (e–h) twenty randomly initialized WTmetaD simulations (i–l) ten randomly initialized WTmetaD simulations and 10 WTmetaD simulations are subject to a two-dimensional harmonic potential localized in CV space [the harmonic potential centers are represented as circles in (j)]. (m) Error of the mean force normalized by the explored CV-space volume as a function of the number of simulation steps. (n) AAD from the analytical FES, normalized by the explored CV-space volume as a function of the number of simulation steps.

The long simulation, depicted in the first row of Figure 3, shows a similar result to the alanine dipeptide example reported in Figure 1c–e. In comparison, the short simulations depicted in the second-row sample less of the configuration space. Given the same simulation and biasing parameters, the short simulations do not have the time to build up a MetaD potential that enables a reversible crossing of the free energy barriers separating two metastable states. However, crucially, at least some of these simulations can undergo a transition. As a result, even if no back and forth *recrossings* are observed in any individual simulation, the transition region is captured with a moderate error, while the relative stability of the metastable states is captured with accuracy comparable to that granted by the long simulation reference (top row of Figure 3). Nonetheless, for a more accurate estimate of the FES, further sampling of the transition region is required, which can be done efficiently by employing other biasing methods.

### 4.2 Combining MetaD with Static Harmonic Potentials

The results presented in the section above exemplify the fairly typical situation in which the convergence of an FES needs to be locally improved in specific regions of CV-space. In such cases, additional simulations can be performed using a combination of biases aimed to locally improve the accuracy of the free energy estimate for configurations mapped in those undersampled regions. To demonstrate this approach, we consider improving the FES calculation for the first ten short simulations from those employed in the section above. In Figure 3f, it can be seen that the region connecting the two basins is poorly sampled. To increase sampling in this region, we perform ten additional short MetaD simulations subject also to a two-dimensional harmonic potential centered in the red circles depicted in Figure 3j. The combination of ten exploratory MetaD and ten MetaD simulations localized in a specific region CV-space leads to the results reported in the third row of Figure 3. As can be observed, the reconstructed FES provides a better estimate of the equilibrium probability not only in the local minima corresponding to metastable states but also for high-free energy, low-probability configurations.

In Figure 3m,n, we report the on-the-fly convergence to measure  side by side with the AAD/*v*. It can be seen that the global convergence of the different sets of simulations can be systematically monitored and compared, quantitatively capturing the improvement introduced by focusing on under-sampled CV regions with time-independent harmonic potentials.

This analysis shows that the flexibility granted by using multiple biasing potentials together with independent short simulations enables to obtain, monitor and improve convergence with independent simulations subject to different biases.

## 5 Applications

In this section, we discuss how the approaches illustrated on simple model systems in the previous sections can be used to monitor and improve the convergence of FESs for complex processes. We focus our attention on modeling nucleation, a task where converging FESs are often limited by the inherent slow dynamics in CV space and where combining multiple simulations enables us to improve our ability to obtain accurate FESs.

### 5.1 Liquid Droplet Nucleation from a Supersaturated Vapor

The first application is the numerical calculation of FESs associated with the nucleation of a liquid droplet from a supersaturated vapor, a rare-event process initiating the condensation of a liquid phase. In this case, a system with 512 argon atoms was simulated under the canonical ensemble at four increasing supersaturation levels. Sampling this process with brute force simulations is extremely impractical and only possible for *billion* atom simulations.^36^ However, even enhanced sampling techniques such as MetaD, while being instrumental in efficiently recovering the kinetics of nucleation,^34^ are rather inefficient at determining the full FES. This is due to the fact that a large, asymmetric, free energy barrier separates the basins corresponding to the metastable parent phase and the stable state. Moreover, the characteristic fluctuations in CV space are orders of magnitude different in the two states. As such, different WTmetaD parameters (such as Gaussian width and bias factor) are desirable for efficiently sampling the forward (condensation) and backward (evaporation) transitions.

**Figure 4:**
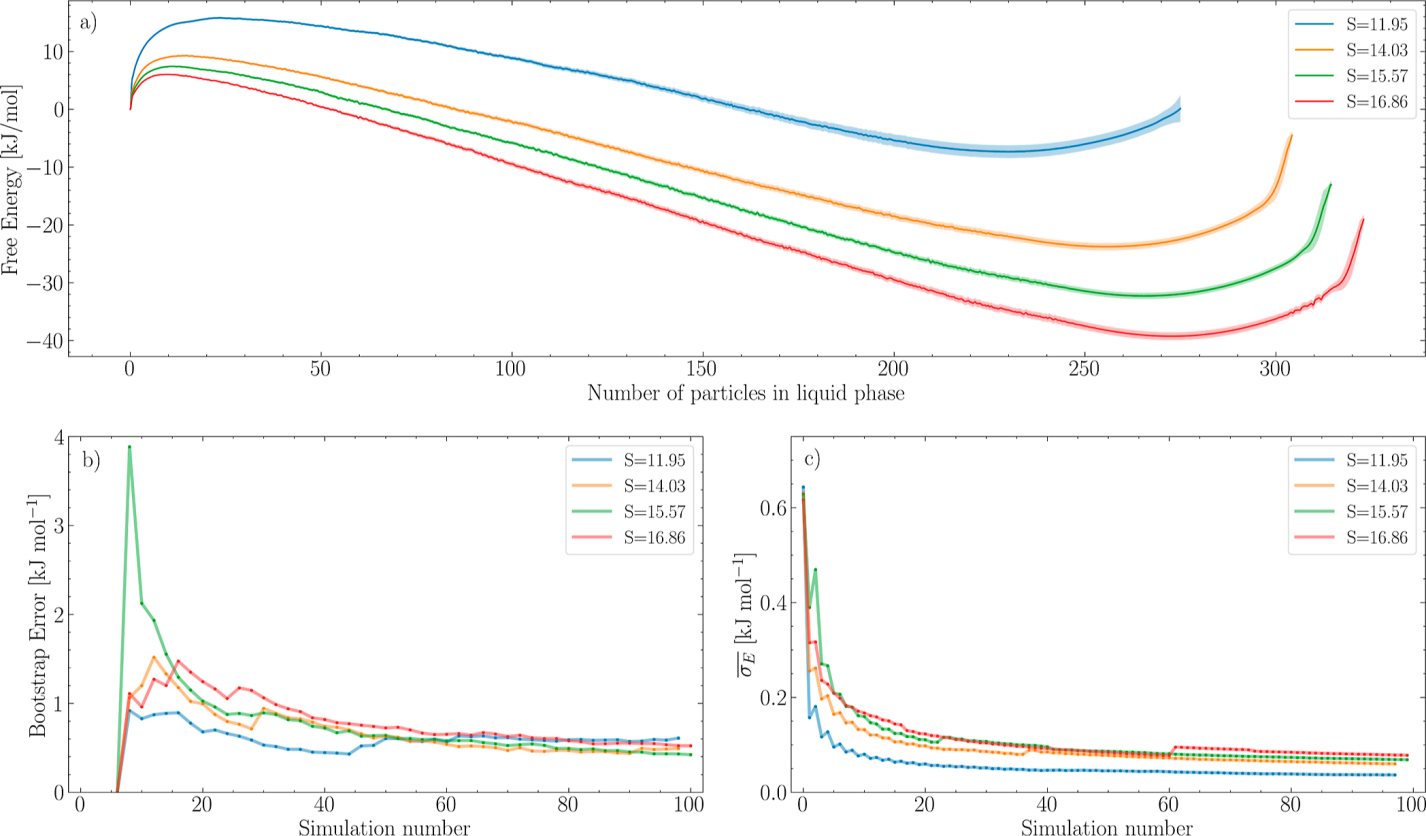
Combining multiple short simulations to estimate the FES associated with the nucleation of a liquid droplet from supersaturated vapor. (a) FES associated with the number of molecules in the liquid phase^34^ (see the Supporting Information for the simulation details) at different supersaturation levels. The shaded region represents the standard deviation calculated with a bootstrap analysis. (b) Progression of the standard deviation of the FES as a function of bootstrap iterations for various levels of supersaturation. (c) Progression of the standard error of the mean force as a function of the number of simulations for various levels of supersaturation.

Here, we show that the biasing strategy can be tailored specifically to this problem by using MFI to postprocess the simulation results. For the forward transition, a WTmetaD bias is applied with narrow hills, whereas for the backward transition, a MetaD bias with wider hills is applied. Moreover, given the asymmetry of the barrier, forward transitions can take place with much smaller bias factors than backward transitions. Additionally, for the lowest supersaturation level, a higher energy barrier is expected, and thus, an additional static bias potential is added to the forward simulation, increasing the efficiency of the construction of the bias potential necessary to observe nucleation events. To adequately sample the whole FES and provide sufficient data sampling configurations that cross the free energy barrier, 50 forward simulations and 50 backward simulations were conducted until the other stable state was reached. This protocol was repeated for various levels of supersaturation. Additional information regarding the simulation setup is reported in the Supporting Information. The forces from all trajectories were calculated and patched together with MFI to find the FES, depicted in Figure 4a, and the convergence was monitored with the on-the-fly error of the mean force, illustrated in Figure 4c. Additionally, a bootstrap analysis was performed on the independent forces, yielding an uncorrelated standard deviation of the FES. That was used as error bars for the FES, and the progression of the global average is presented in Figure 4b. The shape of the FES of the nucleation event is captured well, and the bootstrap analysis indicates a low error in the transition region, whereas the tail of the FES has a larger error.

### 5.2 Two-Step Crystallization of a Colloidal System

To demonstrate the application of the  convergence estimator when deploying a set of independent simulations in a complex application, we analyze the convergence behavior of multiple MetaD calculations modeling a colloidal system undergoing a two-step nucleation process.^37^ The CVs used to describe this process are *n*, counting the number of particles with a coordination number above some threshold, and *n*(*Q*6), counting the number of particles with a local Steinhardt order parameter (*Q*6) above a representative threshold. In such simulations, the CVs are efficiently computed via a graph neural network (GNN) model, which offers orders-of-magnitude gains in computational efficiency in the on-the-fly evaluation of the CVs necessary when conducting biased sampling. Additional information about the GNN method and the simulation details can be found in ref (35) and in the Supporting Information.

**Figure 5:**
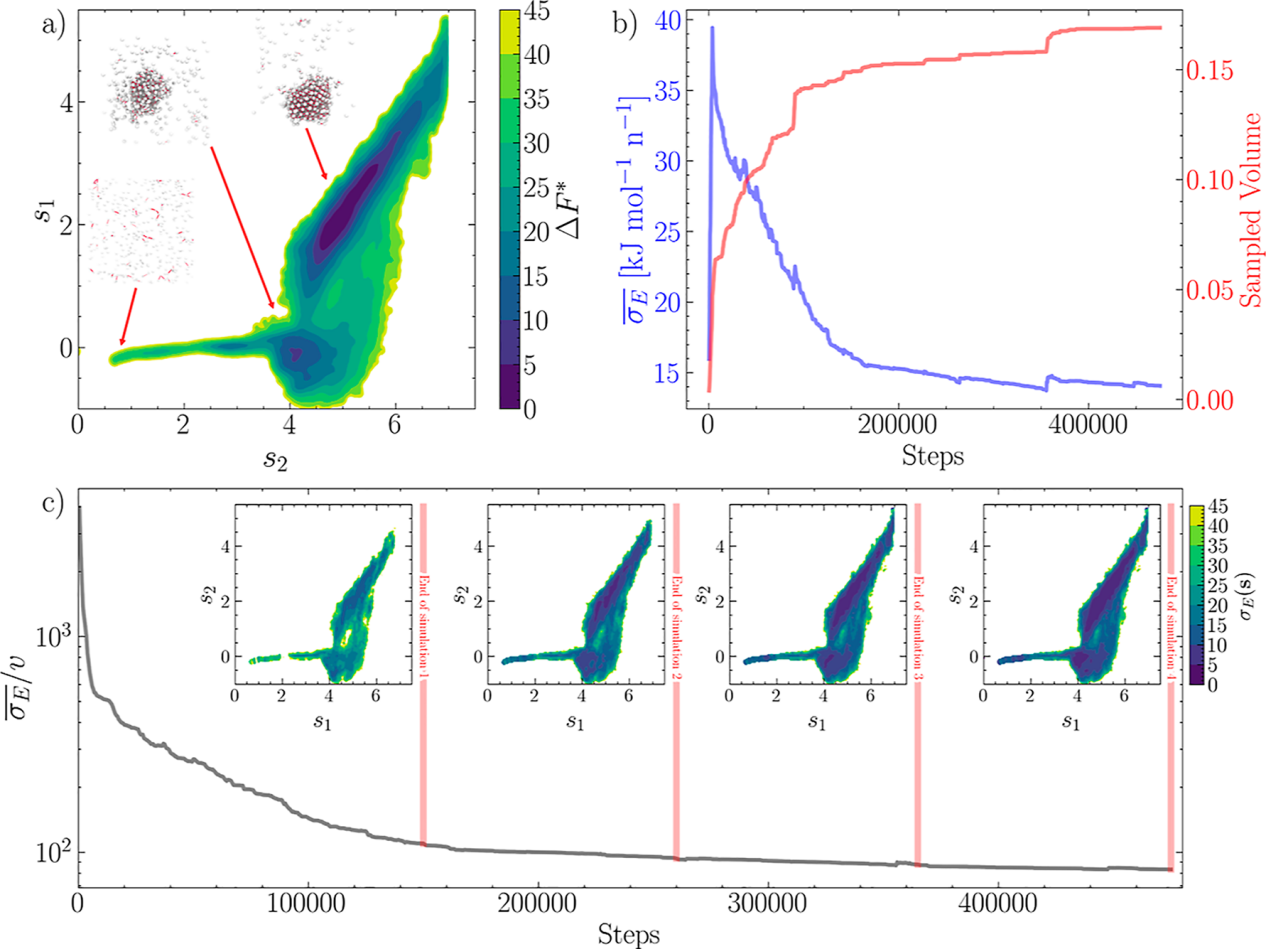
Improving and monitoring the convergence of FES by combining multiple independent WTmetaD simulations. (a) Converged FES (in reduced units) obtained merging four independent WTmetaD and MetaD simulations performed with different biasing parameters. The CVs *s*_1_ and *s*_2_ are GNN-based approximations of nucleation collective variables *n* and *n*(*Q*6) discussed in detail in ref (35). (b) Time series of the configurational volume explored by the simulations (blue) and the CV-space averaged value of the standard error of the mean force in CV space. (c) Global  and local convergence estimators, demonstrating a systematic convergence of the FES associated with providing additional data obtained from independent simulations performed with different biasing setups.

The system modeled consists of 421 particles in a cubic box of length 92.83σ, modeled via a Derjaguin–Landau–Verwey–Overbeek^38−40^ potential. All simulations were performed in the *NVT* ensemble, tempered at 2T*. This investigation entailed four independent MetaD simulations. Three simulations utilized WTmetaD with varying bias factors, while the fourth was a nontempered MetaD simulation. Additional simulation details^41−48^ are reported in the Supporting Information, input files necessary to reproduce the relevant examples are available on PLUMED-NEST.^49^ The configurations sampled across all simulations were postprocessed and combined into a single FES using MFI. The combined FES is shown in Figure 5a, where two main basins can be seen. The deeper basin at large values of both *s*_1_ and *s*_2_ represents configurations embedding a crystalline particle surrounded by a vapor of colloidal particles in dynamic equilibrium with each other. The second basin, at low *s*_2_, represents configurations where a dense liquid droplet is present in the simulation box as a metastable intermediate along the crystallization pathway. These two metastable states are separated by a free energy barrier around *n*(*Q*6) ≈ 1. In Figure 5b, the progression of the on-the-fly error of the mean force and the progression of the sampled volume is shown. While the error  (blue line) shows a decreasing trend, there are occasional upward fluctuations corresponding to an increase in the explored space *v* (red line).

The overall convergent behavior of the four combined simulations is exemplified by the convergence measure , reported in Figure 5c, clearly demonstrating the systematic improvement of the FES obtained by combining self-consistent data generated via independent MetaD simulations. A mapping of σ_E_ in CV space is reported in the insets of Figure 5c, demonstrating that the overall, average convergent behavior of the set of simulations performed here is indeed accompanied by an overall convergent behavior across the entire CV space, and it is not dictated by a local reduction of the error σ_E_(*s*).

## 6 Conclusions

In this work, we developed a measure of the convergence of the sampling of configurational FESs based on MFI and biased dynamics. Recognizing that convergence, in the context of adaptive sampling, refers to the ability to visit new and relevant molecular configurations, as well as the accurate determination of their equilibrium probability, the convergence measure that we propose is the error of the *mean force* in CV space, normalized by a measure of the *volume* explored in CV space. We show that this measure of biased sampling convergence can be computed on-the-fly and correlates strongly with the FES error computed a posteriori via bootstrapping independent simulations or block averaging. In addition to providing a measure of convergence, we show with examples and applications that, postprocessing biased simulations with MFI, one can combine different static and dynamic biases, thereby targeting the convergence of FES in specific regions of CV space with significant flexibility. Combining the sampling obtained under the effect of different biases also enables us to systematically improve on simulations performed with suboptimal setups without discarding data, thus making the most of the often unreported computing time allocated to fine-tune bias simulation parameters. The convergence estimators, as well as different strategies for combining biases, are demonstrated with a range of examples of increasing complexity, including analytical models, conformational changes of alanine dipeptide, the nucleation of a liquid argon droplet from a supersaturated vapor and the nucleation of a colloidal crystal from solution. All examples are implemented via the pyMFI Python library, which is publicly accessible at https://github.com/mme-ucl/pyMFI. Use cases and simple examples of the use of pyMFI to postprocess biased simulations are provided online and in the Supporting Information.

## Data Availability

The input files for the simulations using PLUMED are available at PLUMED NEST under the ID plumID:24.013 (https://www.plumed-nest.org/eggs/24/013/).^49^ The MFI method, implemented in the “pyMFI” Python library, can be accessed at https://github.com/mme-ucl/pyMFI. Instructions for using this library to reproduce the results of this work are provided as Jupyter Notebooks at https://github.com/mme-ucl/MFI.

## Supporting Information Available

Scripts employing the pyMFI library, analysis of the convergence estimator dependence on the kernel bandwidth, and simulation setup details (PDF)
