# Supplementary material for: Estimating Free-Energy Surfaces and Their Convergence from Multiple, Independent Static and History-Dependent Biased Molecular-Dynamics Simulations with Mean Force Integration
Source: J Chem Theory Comput. 2024 Jun 24;20(13):5418–27. doi: 10.1021/acs.jctc.4c00091 (PMC11238544; doi:10.1021/acs.jctc.4c00091)
Supplement: Supplementary file 1 — ct4c00091_si_001.pdf [file ct4c00091_si_001.pdf]

Supplementary Materials: Estimating Free Energy Surfaces and their Convergence from multiple, independent static and history-dependent biased molecular-dynamics simulations with Mean Force Integration.

Antoniu Bjola and Matteo Salvalaglio
Thomas Young Centre and Department of Chemical Engineering, University College London, London, WC1E 7JE, United Kingdom.
(*m.salvalaglio@ucl.ac.uk)

PYMFI EXAMPLES

In this section, the pyMFI library[1] is displayed, and interested readers are encouraged to run the code themselves. First, the one-dimensional analytical surface (illustrated in Figure 1a) is simulated with Langevin dynamics, and the results are analysed with MFI. Next, the same one-dimensional surface is simulated and analysed using different simulation parameters and the results from the two simulations are patched. After that, a two-dimensional analytical surface (illustrated in Figure 2) is simulated and analysed with MFI twice, the first time employing only a metadynamics bias and the second time biasing the simulation with metadynamics and harmonic potential.
Later we report the simulation setup for the one- and two-dimensional system, followed by the setup for the simulations of alanine dipeptide, the nucleation of the supersaturated Argon vapour, and the nucleation of the colloidal system.
The Python code is kept at a level accessible to most readers, but interested readers can have a deeper look at the full library at https://github.com/mme-ucl/MFI and make use of the documentation for a better understanding of the functions. Additional Jupyter notebooks available on GitHub provide additional examples.
Langevin simulations can easily be performed on a personal computer with the PLUMED software [2]. The simulation of molecular systems, such as alanine dipeptide, requires additional software, such as GROMACS[3], to be installed and patched with plumed. However, existing simulations can also be analysed using pyMFI, requiring only the trajectory data represented as CVs and the biasing history (e.g. the COLVAR and HILLS file).

A. 1D Analytical Surface

The first simulation will be performed on a one-dimensional analytical potential presented in figure 1 a), which is defined as $F_{exact}(s) = s^8 - 103e^{-\frac{(s+1.5)^2}{0.07}} - 93e^{-\frac{(s+0.5)^2}{0.05}} - 70e^{-\frac{s^2}{0.03}} + 50e^{-\frac{(s-0.5)^2}{0.03}} - 50e^{-\frac{(s-1)^2}{0.1}}$. It will be simulated with a Langevin dynamics simulation with a Metadynamics bias. The trajectory data represented as CVs (name of file: “position”) and the biasing history (name of file: “HILLS”) is analysed with MFI to determine the force-terms (i.e. average mean force, probability density, standard error of the mean force, and other relevant results). Lastly, the average mean force is integrated to obtain the FES, and the results are shown in figure S1.

```
from pyMFI import MFI1D
from pyMFI import run_plumed


#Simulation steps and analytical function as string
n_steps = 2000000
f = "1*x^8-50*exp(-(x-1)^2/0.1)-93*exp(-(x+0.5)^2/0.05)-103*exp(-(x+1.5)^2/0.07)-70*exp
    (-(x)^2/0.03)+50*exp(-(x-0.5)^2/0.03)"

#Run Metadynamics simulation on custom potential
run_plumed.run_langevin1D(analytical_function=f, temperature=1, simulation_steps=n_steps
    ,gaus_width=0.1, gaus_height=2.5, biasfactor=20, gaus_pace=100)

#Read the HILLS file
HILLS = MFI1D.load_HILLS(hills_name="HILLS")

#Read the COLVAR File
position = MFI1D.load_position(position_name="position")
```

h

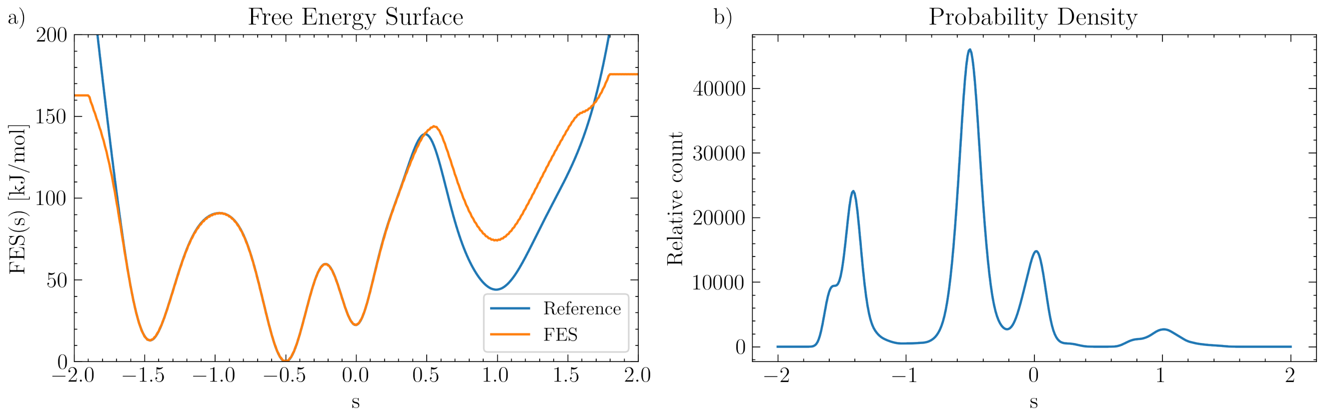

FIG. S1. a) MFI estimate of a monodimensional multi-well free energy surface obtained postprocessing a single metadynamics simulation (orange line) with the exact FES (blue line). b) MFI estimate of the probability density of single metadynamics simulation.

```
#Compute the time-independent mean force
results = MFI1D.MFI_1D(HILLS=HILLS, position=position, bw=0.03, kT=1, log_pace=4000,
    error_pace=200, min_grid=-2, max_grid=2, nbins=401, WellTempered=1,
    use_weighted_st_dev=False)
X, Ftot_den, Ftot_den2, Ftot, ofv_num, FES, ofv, ofe, cutoff, error_evol,
    fes_error_cutoff_evol = results

#integration on a non-periodic domain
FES = MFI1D.intg_1D(Ftot, X[1]-X[0])
```

B. Combining Two Simulations

Having performed and analysed the first simulation, the force terms are saved so that they can later be combined with the next simulations. Next, an additional simulation is conducted on the same system, this time changing some parameters such that the simulation starts in the basin on the right and the metadynamics of Gaussian hills are higher. After the simulation, the trajectory and biasing history are analysed with MFI, combined with the previous force terms, and the Mean Force is integrated to find the FES. The results are shown in figure S2.

```
#Save relevant terms of the last simulation: [Sum of the Probability Density, Sum of the
    Squared Probability Density, Average Mean Force, Denominator of the Variance of the
    Mean Force]
force_terms = [[Ftot_den, Ftot_den2, Ftot, ofv_num]]

#Run Metadynamics simulation on custom potential with new parameters
run_plumed.run_langevin1D(analytical_function=f, initial_position=1.0, temperature=1,
    simulation_steps=n_steps ,gaus_width=0.1, gaus_height=5, biasfactor=50, gaus_pace=100)

#Read the HILLS and COLVAR file
HILLS = MFI1D.load_HILLS(hills_name="HILLS")
position = MFI1D.load_position(position_name="position")

#Compute the time-independent mean force
results = MFI1D.MFI_1D(HILLS=HILLS, position=position, bw=0.03, kT=1, log_pace=4000,
    error_pace=200, min_grid=-2, max_grid=2, nbins=401, WellTempered=1,
    use_weighted_st_dev=False)
X, Ftot_den, Ftot_den2, Ftot, ofv_num, FES, ofv, ofe, cutoff, error_evol,
    fes_error_cutoff_evol = results
```

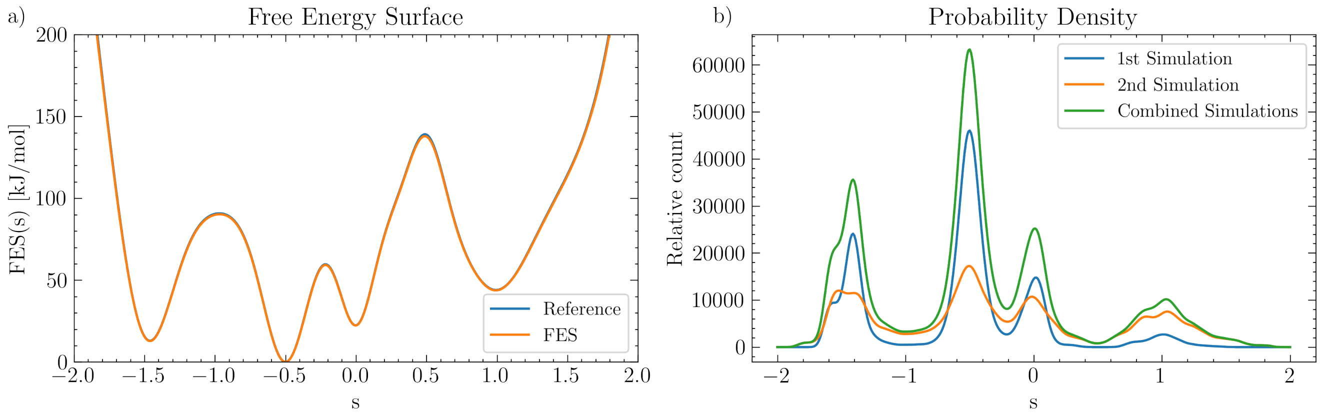

FIG. S2. a) MFI estimate of a monodimensional multi-well free energy surface obtained post-processing two metadynamics simulations (orange line) with the exact FES (blue line). b) MFI estimate of the probability density of first metadynamics simulation (blue line), second metadynamics simulation (orange line), and combined metadynamics simulations (green line)

```
#Combine the two results
force_terms.append([Ftot_den, Ftot_den2, Ftot, ofv_num])
Ftot_den_patch, Ftot_den2_patch, Ftot_patch, ofv_num_patch, ofe, Aofe_progression = MFI1D
    .patch_forces_ofe(np.asarray(force_terms), use_weighted_st_dev=False)

#integration on a non-periodic domain
FES = MFI1D.intg_1D(Ftot, X[1]-X[0])
```

C. 2D Analytical Surface

The next simulation will be performed on the two-dimensional double well analytical potential presented in figure 3 a), which is defined as $F_{exact}(s_1, s_2) = 1.35s_1^4 + 1.90s_1^3s_2 + 3.93s_1^2s_2^2 - 6.44s_1^2 - 1.90s_1s_2^3 + 5.59s_1s_2 + 1.33s_1 + 1.35s_2^4 - 5.56s_2^2 + 0.90s_2 + 18.59$. The results are shown in figure S3.

```
#Simulation steps and analytical function
n_steps = 1000000
grid = np.linspace(-3, 3, 200)
X, Y = np.meshgrid(grid, grid)
inve_pot = 1.34549*X**4+1.90211*X**3*Y+3.92705*X**2*Y**2-6.44246*X**2-1.90211*X*Y
    **3+5.58721*X*Y+1.33481*X+1.34549*Y**4-5.55754*Y**2+0.904586*Y+18.5598
inve_pot = inve_pot - np.min(inve_pot)
f = "1.34549*x^4+1.90211*x^3*y+3.92705*x^2*y^2-6.44246*x^2-1.90211*x*y^3+5.58721*x*y
    +1.33481*x+1.34549*y^4-5.55754*y^2+0.904586*y+18.5598"

#Run Metadynamics simulation on custom potential
run_plumed.run_langevin2D(simulation_steps=n_steps, analytical_function=f, gaus_width_x
    =0.1, gaus_width_y=0.1, gaus_height=0.5, biasfactor=10, gaus_pace=500, file_extension=
    "_2D")

#Read the HILLS file
HILLS=MFI.load_HILLS_2D(hills_name="HILLS_2D")

#Read the Colvar File
[position_x, position_y] = MFI.load_position_2D(position_name="position_2D")

#COMPUTE Mean force and weight of the simulation
results = MFI.MFI_2D(HILLS=HILLS, position_x=position_x, position_y=position_y, bw=np.
    array((0.05, 0.05)), kT=1, min_grid=np.array((-3, -3)), max_grid=np.array((3, 3)),
    error_pace=20, ref_fes=inve_pot, use_weighted_st_dev=False)
```

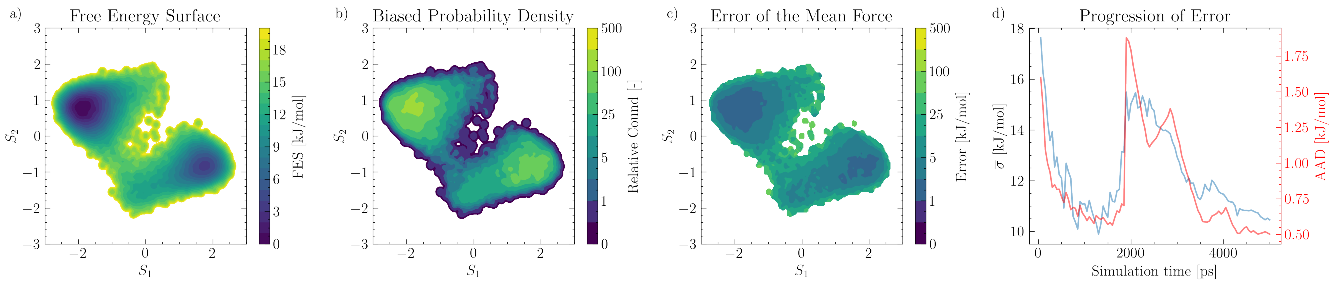

FIG. S3. Results obtained post-processing a Langevin Dynamic simulation of a two-dimensional double-well system biased with MetaD. a) MFI estimate of the FES. b) Biased probability density. c) Error of the mean force. d) Progression of the error of the mean force (blue line) and progression of the average absolute deviation to the analytical surface (red line)

```
[X, Y, Ftot_den, Ftot_x, Ftot_y, ofv, ofe, cutoff, volume_history, ofe_history,
    aad_history, time_history, Ftot_den2, ofv_num_x, ofv_num_y] = results

#integration on a non-periodic domain
[X, Y, FES] = MFI.FFT_intg_2D(Ftot_x, Ftot_y, min_grid=np.array((-3, -3)), max_grid=np.
    array((3, 3)))
```

Having analysed the simulation on the two-dimensional double well, one can see in figure S3 b) that the transition region is not sampled very well, and the error of the mean force, depicted in figure S3 c), is relatively high in the transition region. Consequently, one might want to sample the transition region more extensively. In that case, a harmonic potential is applied in the centre ($x, y = -0.5, -0.5$, with $\kappa_x = \kappa_y = 40kJ/mol$) while also employing MetaD, to provide a consistent sampling of that region. Similar to the one-dimensional example, the second simulation is analysed, and the force terms of both simulations are combined.
It is possible to use the optional argument "base_terms" in the MFI_2D function, which requires the force terms of a previous simulation as input. If this option is used, the on-the-fly error calculated is directly patched with the force terms specified in "base_terms", yielding the combined error. Also, the ofe_history of both simulations can be merged, resulting in a continuous error progression. However, the force terms returned by the MFI_2D function result from the "HILLS" and "position" data and need to be patched with the other force terms using the patch_2D function.

```
#Save force terms from first simulation
force_terms_2D = Ftot_den, Ftot_den2, Ftot_x, Ftot_y, ofv_num_x, ofv_num_y
history_terms = volume_history, ofe_history, aad_history, time_history

#Run Metadynamics simulation on custom potential
n_steps = 1000000
run_plumed.run_langevin2D(simulation_steps=n_steps, analytical_function=f, gaus_width_x
    =0.05, gaus_width_y=0.05, gaus_height=0.2, biasfactor=5, gaus_pace=500, hp_centre_x
    =-0.5, hp_centre_y=-0.5, hp_kappa_x=40, hp_kappa_y=40, file_extension="_HP")


#Read the HILLS and Colvar File
HILLS=MFI.load_HILLS_2D(hills_name="HILLS_HP")
[position_x, position_y] = MFI.load_position_2D(position_name="position_HP")

#COMPUTE Mean force and weight of the simulation
results = MFI.MFI_2D(HILLS = HILLS, position_x = position_x, position_y = position_y, bw
    = np.array((0.05, 0.05)), kT = 1, min_grid=np.array((-3, -3)), max_grid=np.array((3,
    3)), error_pace = 20, ref_fes=inve_pot, base_terms=force_terms_2D, hp_centre_x=-0.5,
    hp_centre_y=-0.5, hp_kappa_x=40, hp_kappa_y=40, use_weighted_st_dev = False)
[X, Y, Ftot_den, Ftot_x, Ftot_y, ofv, ofe, cutoff, volume_history, ofe_history,
    aad_history, time_history, Ftot_den2, ofv_num_x, ofv_num_y] = results

#Patch the two simulations and the error history
Ftot_den_patch, Ftot_den2_patch, Ftot_x_patch, Ftot_y_patch, ofv_num_x_patch,
    ofv_num_y_patch = MFI.patch_2D([force_terms_2D, [Ftot_den, Ftot_den2, Ftot_x, Ftot_y,
    ofv_num_x, ofv_num_y]])
```

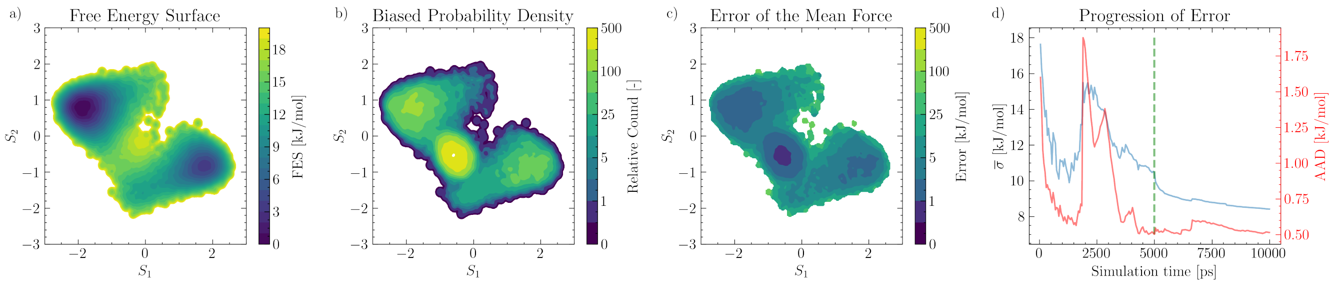

FIG. S4. Results obtained post-processing two Langevin Dynamic simulation of a two-dimensional double-well system. The former simulation was biased with MetaD (results shown in figure S3 and the latter biased with MetaD and US. a) MFI estimate of the FES. b) Biased probability density. c) Error of the mean force. d) Progression of the error of the mean force (blue line) and progression of the average absolute deviation to the analytical surface (red line)

```
ofe_history = history_terms[1] + ofe_history
aad_history = history_terms[2] + aad_history
time_history = history_terms[3] + [e+history_terms[3][-1] for e in time_history]

#integration on a non-periodic domain
[X, Y, FES] = MFI.FFT_intg_2D(Ftot_x_patch, Ftot_y_patch, min_grid=np.array((-3, -3)),
    max_grid=np.array((3, 3)))
```

S1. CONVERGENCE ESTIMATOR

The convergence estimator introduced in section III. measures the statistical variance (or standard error) of the mean force. By following its progression, one can better understand which regions of the CV-space have converged, and which regions require further sampling. However, the calculation of the mean force (see Eq. 2 and Eq. 3) depends on the probability density (see Eq. 4), which in turn depends on h, the bandwidth of the Gaussian kernel. Therefore, the choice of the bandwidth affects the standard error of the mean force. Choosing a bandwidth that is too small results in a noisy probability density, leading to a noisy estimation of the mean force and the free energy surface. Consequently, the standard error of the mean force tends to be higher with lower bandwidths. Conversely, a bandwidth that is too large, will result in a probability density that is excessively smooth, impairing its ability to capture local changes in sampling accurately. However, this smoothing leads to a smoother average mean force, and thus, a lower standard error of the mean force.

Nonetheless, the convergence estimation does not provide an accurate error, but rather a qualitative assessment of the estimate of the average mean force. A lower bandwidth will result in a higher standard error of the mean force and vice versa, but the qualitative trend in the progression of the standard error remains consistent, as shown in Figure S5. Ultimately, the optimal choice for the bandwidth depends on the roughness of the free energy surface, and the amount of sampling available for the estimation of the probability density.

S2. ATOMISTIC WT-METAD SIMULATION DETAILS.

A. Alanine Dipeptide

The conformational structures of alanine dipeptide in vacuo were obtained from molecular dynamics simulations under the effect of a well-tempered metadynamics bias, with the two backbone torsion angles Ψ and Φ as collective variables. The molecular dynamics simulations were performed with GROMACS[3] 2021 patched with PLUMED[2] 2.7.5, using the AMBER99SB force field[4]. The system consists of one alanine dipeptide molecule, simulated in a box of size $2.86 \times 2.86 \times 2.02$ nm with no periodic boundaries. Prior to the production run, energy minimization was performed with a steepest descent algorithm with a residual force tolerance of 10 $kJ \times mol^{-1} nm^{-1}$. A cut-off of 1.2 nm for non-bonded interactions was chosen, long-range intermolecular interactions are accounted for using the Particle Mesh Ewald (PME) approach and all bonds are constrained using the LINCS algorithm. The molecular dynamics simulation

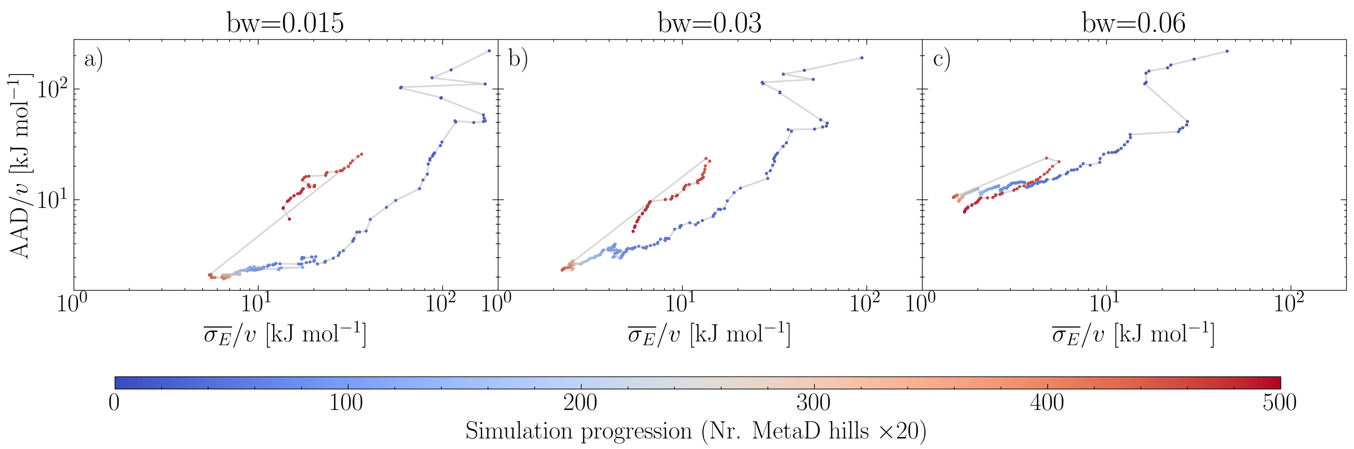

FIG. S5. Comparing the progression of the standard error of the mean force $\overline{\sigma_E}$ divided by the sampled volume v(x-axis) with the average absolute deviation divided by the sampled volume (y-axis). The error progressions in panel a) are calculated with a bandwidth, h, of 0.015. In panel b) a bandwidth of 0.03 was used and in panel c) a bandwidth of 0.06. The blue-red color shift indicates simulation progression.

was executed under NVT conditions (canonical ensemble) at 300K, using a modified Berendsen thermostat (V-rescale thermostat) with a leapfrog integrator and a time step of 2 fs for a total simulation time of 40 ns.

The well-tempered metadynamics bias was constructed by adding a Gaussian hill every 2 ps, with a height of 0.5 kJ/mol, a bandwidth of 0.2 for both Ψ and Φ, and a factor of 5.

Another simulation was performed under the same conditions for 500 ns. This simulation time was long enough that the resulting FES was fully converged and used as a reference surface for the calculation of the average absolute deviation.

The input files for these simulations are available on PLUMED-NEST.

B. Argon Condensation

TABLE S1. Argon condensation simulation setup parameters.

Label	S	l (nm)	p (bars)	δ	ω_0 (kJ/mol)	Δt (ps)	γ
S_1	11.95	15.93	1.27	0.25	0.0075	25	5
S_2	14.03	14.60	1.49	0.25	0.005	25	5
S_3	15.57	14.04	1.66	0.25	0.005	25	5
S_4	16.86	13.62	1.79	0.25	0.005	25	5

The forward and backward transitions of a supersaturated Argon vapour to a liquid droplet were simulated multiple times with GROMACS 4.6.3 patched with PLUMED[2] 2.0. The system consists of 512 argon atoms, and their interactions were described by a Lennard-Jones potential with $\epsilon = 0.99797$ kJ/mol and $\sigma = 0.3405$ nm.

The Lennard-Jones potential was truncated at the cutoff length of 5 σ. The molecular dynamics simulation was executed under NVT conditions (canonical ensemble) at a constant temperature of 72K, controlled using the Bussi-Donadio-Parrinello thermostat[5]. The time step for the integration of the equations of motion was set to 5 fs, and the simulation was allowed to run until the energy barrier was crossed and the other metastable state was sampled. This setup was conducted 50 times for the forward transition (condensation) and 50 times for the backward transition (evaporation) and repeated for supersaturation levels of 11.95, 14.03, 15.57 and 16.86, which was set by specifying the box dimension l, shown in table S1. Table S1 also includes the initial pressure p, the width of the deposited Gaussians δ, their initial height ω_0, the deposition stride of the Gaussians Δt, and the biasfactor γ. For the lowest level of supersaturation ($S_1 = 11.95$), a large and steep energy barrier was expected so that an additional wall potential was used for the forward transition.

C. Two-step Colloidal Model Nucleation

Molecular dynamics simulations of the colloidal system were performed in the canonical ensemble, tempered to 2T* with 421 particles in a cubic box of length 92.83σ using LAMMPS [6]. The colloidal particles are modelled via a Derjaguin-Landau-Verwey-Overbeek (DLVO) potential [7–9] with a cutoff of 12.5σ. Details of the potential, its expected thermodynamic behaviour, and additional simulation details are available to reference [10].
The n and n(Q6) CVs are used to describe the nucleation mechanisms of the colloidal system, which were approximated with a graph neural network. Out of the four simulations, three were biased with WTmetaD, each employing a different bias factor (γ_1=40, γ_1=50, γ_1=60), while the fourth simulation was biased with non-tempered metaD. The PLUMED input files are available on PLUMED-NEST (https://www.plumed-nest.org/, plumID:23.026).
All machine learning models were trained using the NNucleate package, which is built on top of PyTorch [11]. It utilizes functionalities from the MDTraj [12] and MDAnalysis [13, 14] packages to train CVs, augment and manage datasets, analyze models, and translate models into PLUMED-readable CV files. These Python scripts are used as CVs through the PLUMED2 fork PyCV [15]. Converting the models into a format supported by PyCV involves Alphabet's Jax and Flax packages, and the necessary gradients are obtained using Jax's autodifferentiation implementation [16, 17].

[1] Bjola A. and Salvalaglio M. pymfi, 2023.

[2] Bonomi M., Bussi G., Camilloni C., Tribello G., Banáš P., Barducci A., Bernetti M., Bolhuis P., Bottaro S., Branduardi D., and et al. Promoting transparency and reproducibility in enhanced molecular simulations. *Nat. Methods*, 16:670–673, 7 2019.

[3] Lindahl V., Abraham M., Hess B., and van der Spoel D. Gromacs 2021.5 manual. *GROMACS*, 1 2022.

[4] Wang J., Wolf R., Caldwell J., Kollman P., and Case D. Development and testing of a general amber force field. *J. Comput. Chem.*, 25:1157–1174, 2004.

[5] Bussi G., Donadio D., and Parrinello M. Canonical sampling through velocity rescaling. *J. Chem. Phys.*, 126, 2007.

[6] Thompson A., Aktulga H., Berger R., Bolintineanu D., Brown W., Crozier P., Veld P., Kohlmeyer A., Moore S., and et al T., Nguyen. Lammps - a flexible simulation tool for particle-based materials modeling at the atomic, meso, and continuum scales. *Comput. Phys. Commun.*, 271, 2 2022.

[7] Derjaguin B. and Landau L. The theory of stability of highly charged lyophobic sols and coalescence of highly charged particles in electrolyte solutions. *Acta Physicochim. URSS*, 14:58, 1941.

[8] Verwey E. and Overbeek J. *Theory of the Stability of Lyophobic Colloids: The Interaction of Sol Particles Having an Electric Double Layer.* Elsevier, 1962.

[9] Loeb A., Overbeek J., Wiersema P., and King C. The electrical double layer around a spherical colloid particle. *J. Electrochem. Soc.*, 108:269, 1961.

[10] Finney A. and Salvalaglio M. A variational approach to assess reaction coordinates for two-step crystallization. *J. Chem. Phys.*, 158:56–80, 3 2023.

[11] Paszke A., Gross S., Massa F., Lerer A., Bradbury J., Chanan G., Killeen T., Lin Z., Gimelshein N., Antiga L., and et al. Pytorch: An imperative style, high-performance deep learning library. In H Wallach, H Larochelle, A Beygelzimer, F d Alché-Buc, E Fox, and R Garnett, editors, *Advances in Neural Information Processing Systems 32 (NeurIPS 2019)*, volume 32. Curran Associates, Inc., 2019.

[12] McGibbon R., Beauchamp K., Harrigan M., Klein C., Swails J., Hernández C., Schwantes C., Wang L., Lane T., and Pande V. Mdtraj: A modern open library for the analysis of molecular dynamics trajectories. *Biophys. J.*, 109:1528–1532, 10 2015.

[13] Michaud-Agrawal N., Denning E., Woolf T., and Beckstein O. Mdanalysis: A toolkit for the analysis of molecular dynamics simulations. *J. Comput. Chem.*, 32:2319–2327, 7 2011.

[14] Mdanalysis: A python package for the rapid analysis of molecular dynamics simulations, 2016.

[15] Giorgino T. Pycv: a plumed 2 module enabling the rapid prototyping of collective variables in python. *J. Open Source Software*, 4:1773, 10 2019.

[16] Bradbury J., Frostig R., Hawkins P., Johnson M., Leary C., Maclaurin D., Necula G., Paszke A., VanderPlas J., and et al S., Wanderman-Milne. Jax: composable transformations of python +numpy programs. `http://github.com/google/jax`, 2018. Accessed: 2023-08-18.

[17] Heek J., Levskaya A., Oliver A., Ritter M., Rondepierre B., Steiner A., and van Zee M. Flax: A neural network library and ecosystem for jax. `http://github.com/google/flax`, 2020. Accessed: 2023-08-18.
